# Supplementary material for: Lacking mechanistic disease definitions and corresponding association data hamper progress in network medicine and beyond
Source: Nat Commun. 2023 Mar 25;14:1662. doi: 10.1038/s41467-023-37349-4 (PMC10039912; doi:10.1038/s41467-023-37349-4)
Supplement: Supplementary file 1 — Supplementary Information [file 41467_2023_37349_MOESM1_ESM.pdf]

Supplementary Information

Lacking mechanistic disease definitions and corresponding association data hamper progress in network medicine and beyond

Sepideh Sadegh, James Skelton, Elisa Anastasi, Andreas Maier, Klaudia Adamowicz, Anna Möller, Nils M. Kriege, Jaanika Kronberg, Toomas Haller, Tim Kacprowski, Anil Wipat, Jan Baumbach, David B. Blumenthal

Supplementary figures

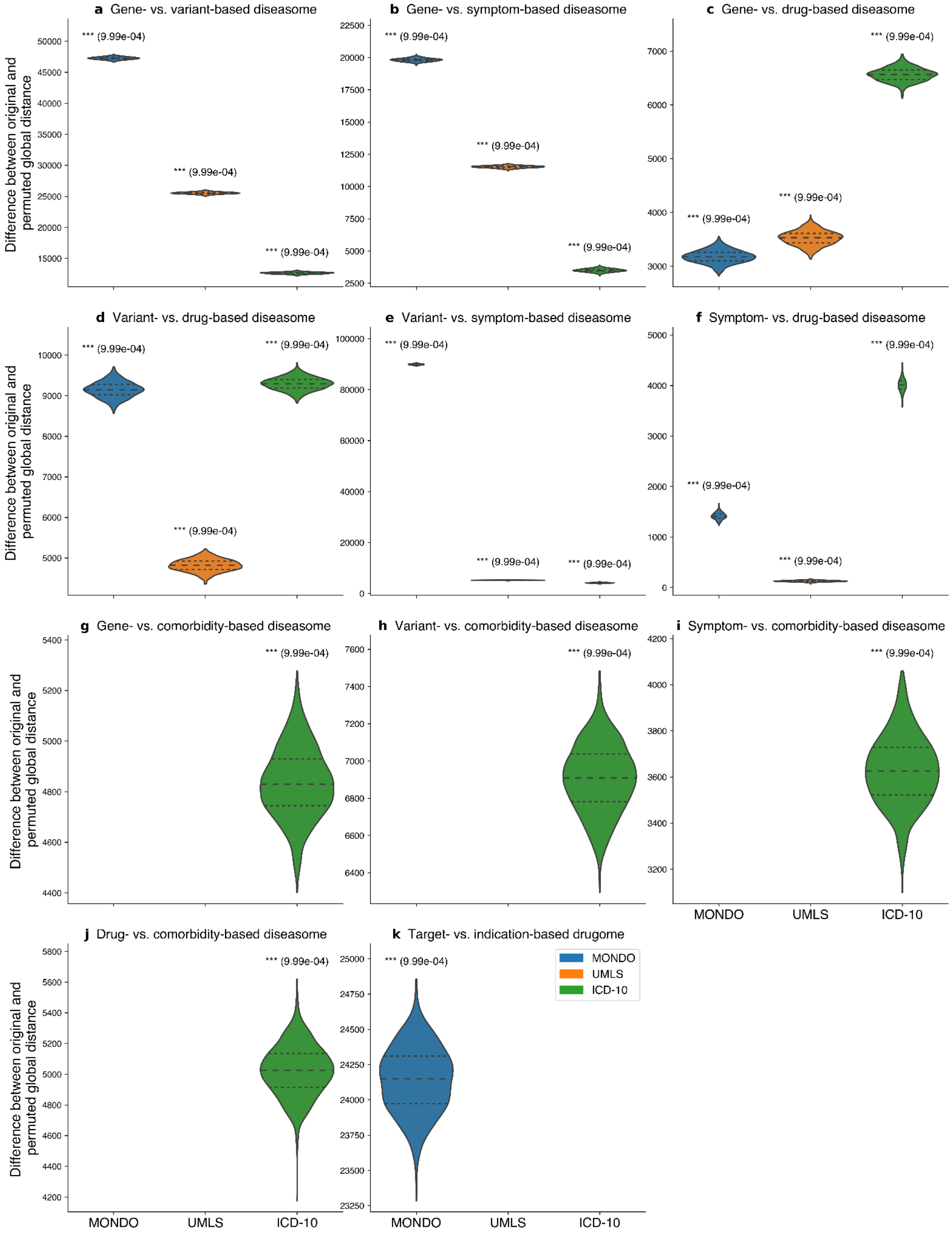

Supplementary Figure 1. Pairwise similarities between networks, global distances, rank-based edge edit costs. Differences of global GEDs between the original and permuted network, and corresponding global empirical *P*-values (one-sided, unadjusted). (a-f) Similarity between diseasomes in MONDO, UMLS CUI and ICD-10 vocabularies. (g-j) Comorbidity-based vs. other diseasomes in ICD-10 vocabularies. (k) Target- vs. indication-based drugomes. All obtained global empirical *P*-values were at the lower resolution limit of our permutation tests with 1,000 randomized network pairs.

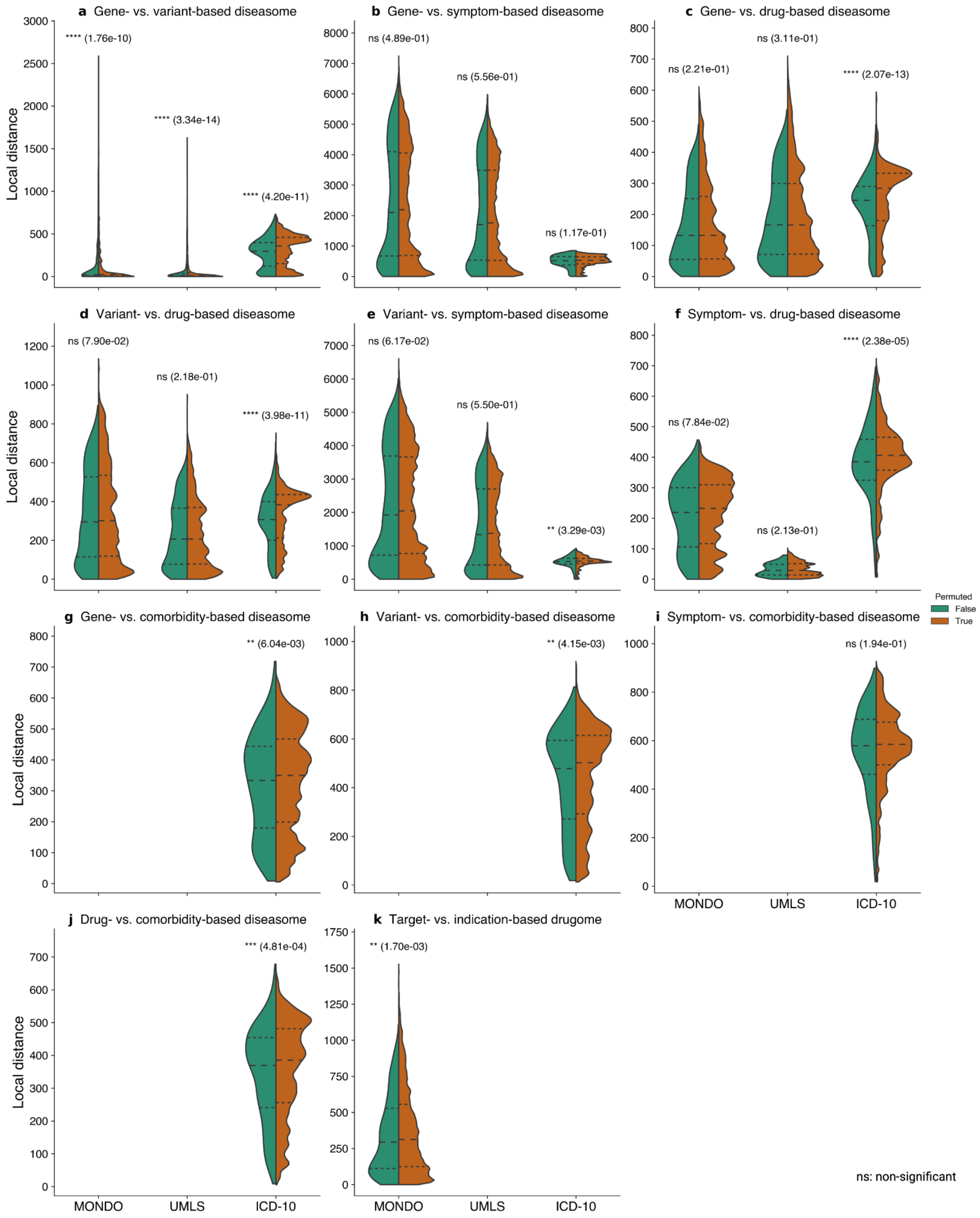

Supplementary Figure 2. Pairwise similarities between networks, global view on local distances, rank-based edge edit costs. Local GEDs (of all nodes) between a pair of networks for the original vs. permuted network and corresponding Mann-Whitney U *P*-values (one-sided, unadjusted). (a-f) Similarity between diseasomes in MONDO, UMLS CUI, and ICD-10 vocabularies. (g-j) Comorbidity-based vs. other diseasomes in ICD-10 vocabulary. (k) target- vs. indication-based drugomes.

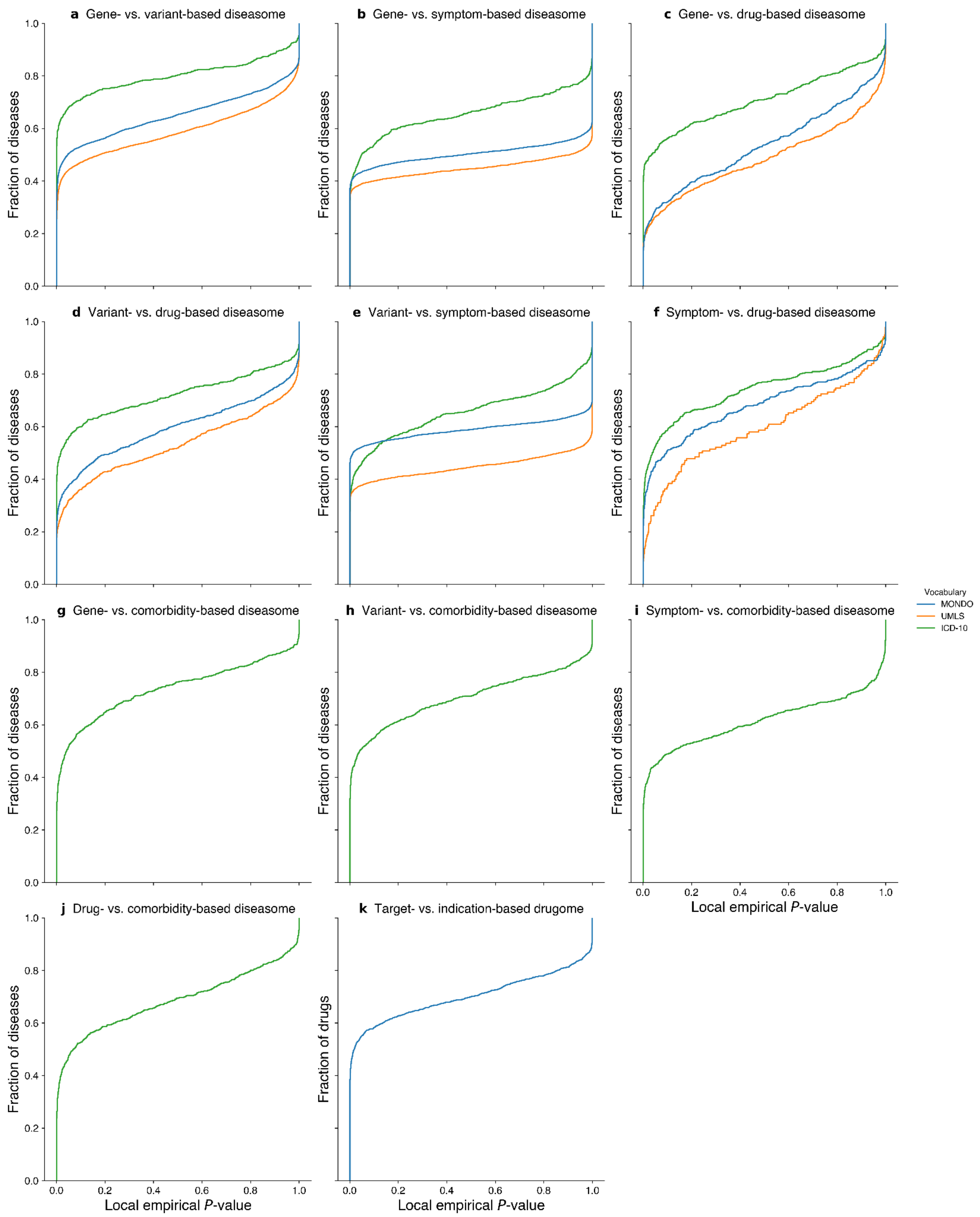

Supplementary Figure 3. Pairwise similarities between networks, local view on local distances, rank-based edge edit costs. Local empirical *P*-values (one-sided, unadjusted) computed from GEDs on a pair of networks for the original vs. permuted network. (a-f) Similarity between diseasomes in MONDO, UMLS CUI, and ICD-10 vocabularies. (g-j) Comorbidity-based vs. other diseasomes in ICD-10 vocabularies. (k) Target- vs. indication-based drugomes.

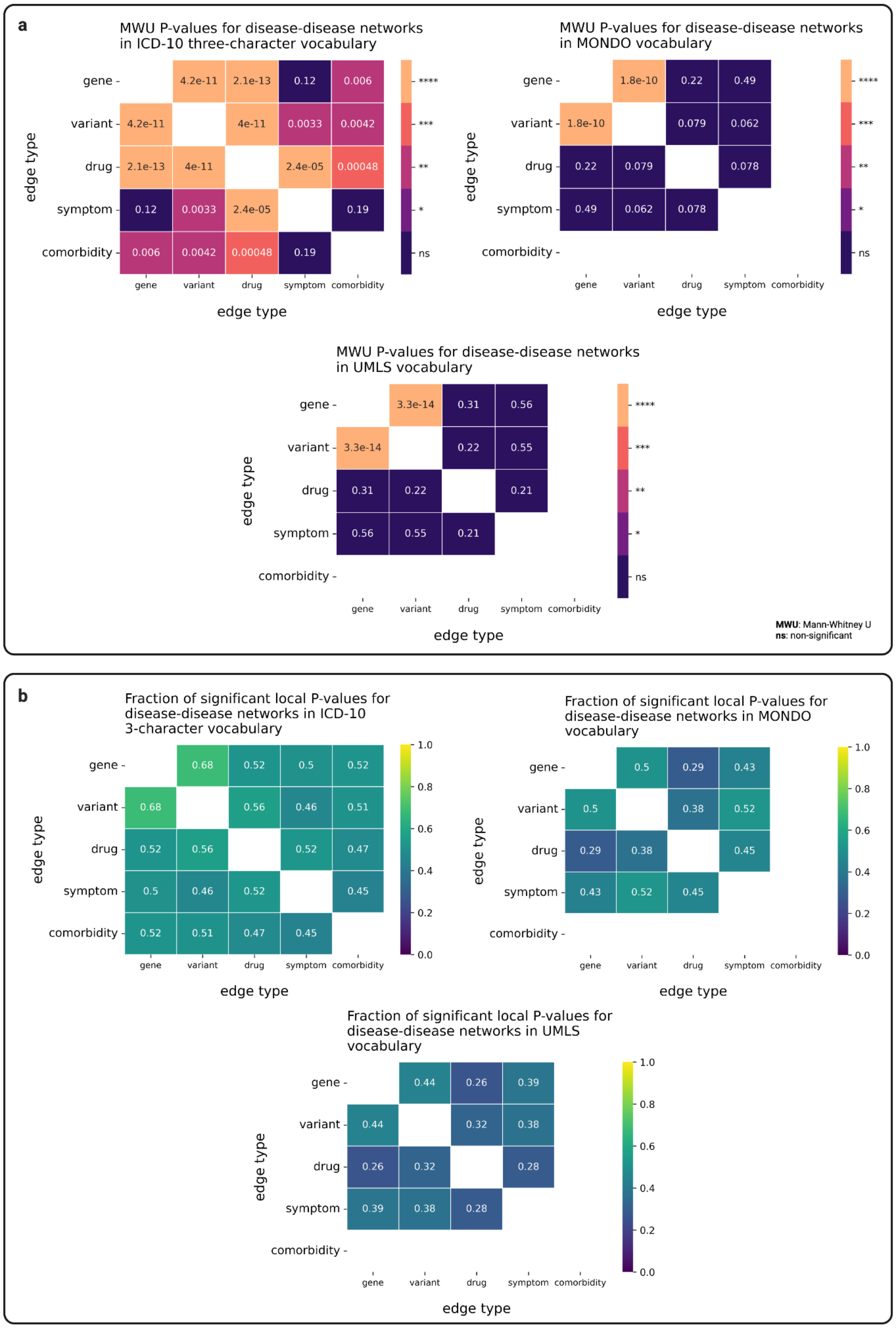

Supplementary Figure 4. Overview of local-scale analyses, rank-based edge edit costs. (a) Mann-Whitney U *P*-values (one-sided, unadjusted) computed from local GEDs with the level of their significance. (b) Fraction of significant local empirical *P*-values (one-sided, unadjusted) at 0.05 level computed from local GEDs on a pair of networks for the original vs. permuted network.

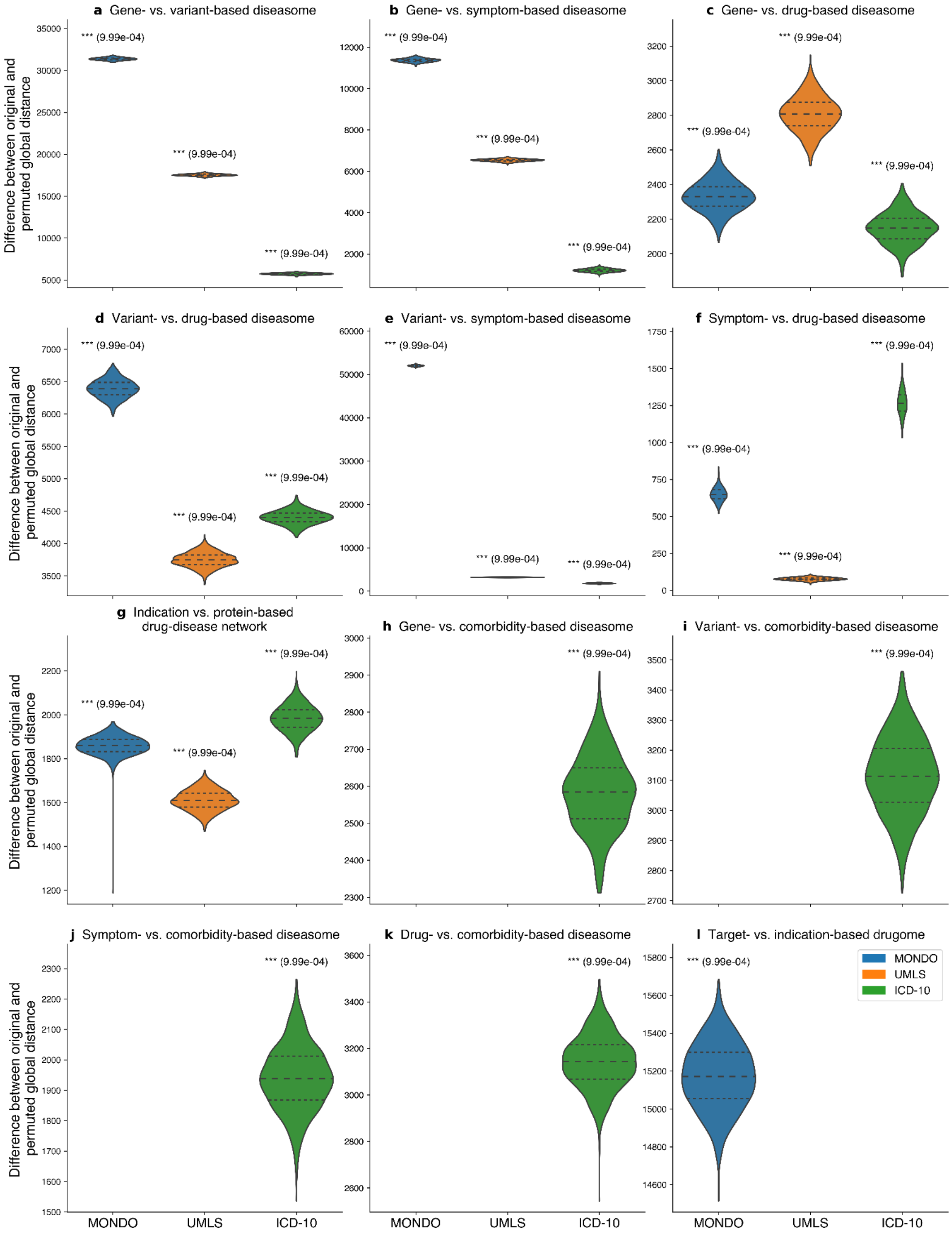

Supplementary Figure 5. Pairwise similarities between networks, global distances, uniform edge edit costs. Differences of global GEDs between the original and permuted network, and corresponding global empirical *P*-values (one-sided, unadjusted). (a-f) Similarities between diseasomes in MONDO, UMLS CUI, and ICD-10 vocabularies. (g) Indication- vs. protein-based drug-disease network in MONDO, UMLS CUI, and ICD-10 vocabularies. (h-k) Comorbidity-based vs. other diseasomes in ICD-10 vocabulary. (l) Target- vs. indication-based drugomes. All obtained global empirical *P*-values were at the lower resolution limit of our permutation tests with 1,000 randomized network pairs.

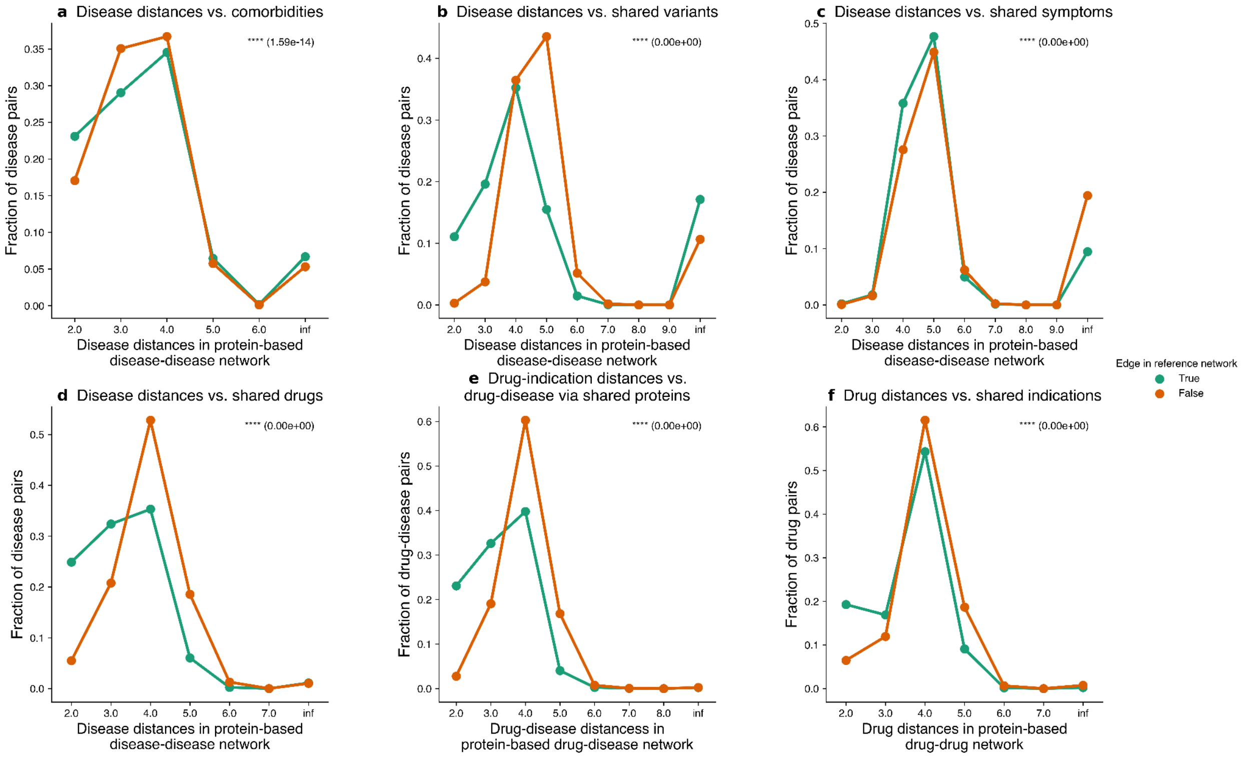

Supplementary Figure 6. Shortest path distances for node pairs with and without edges in reference networks and the corresponding Mann-Whitney U *P*-values (one-sided, unadjusted). (a-d) Disease distances in protein-based disease-disease network vs. different diseasomes as the reference network. (e) Drug-disease distances in protein-based drug-disease network vs. drug-indication network as the reference network. (f) Drug distances in protein-based drug-drug network vs. indication-based drugome as the reference network. All networks are constructed in the MONDO vocabulary.

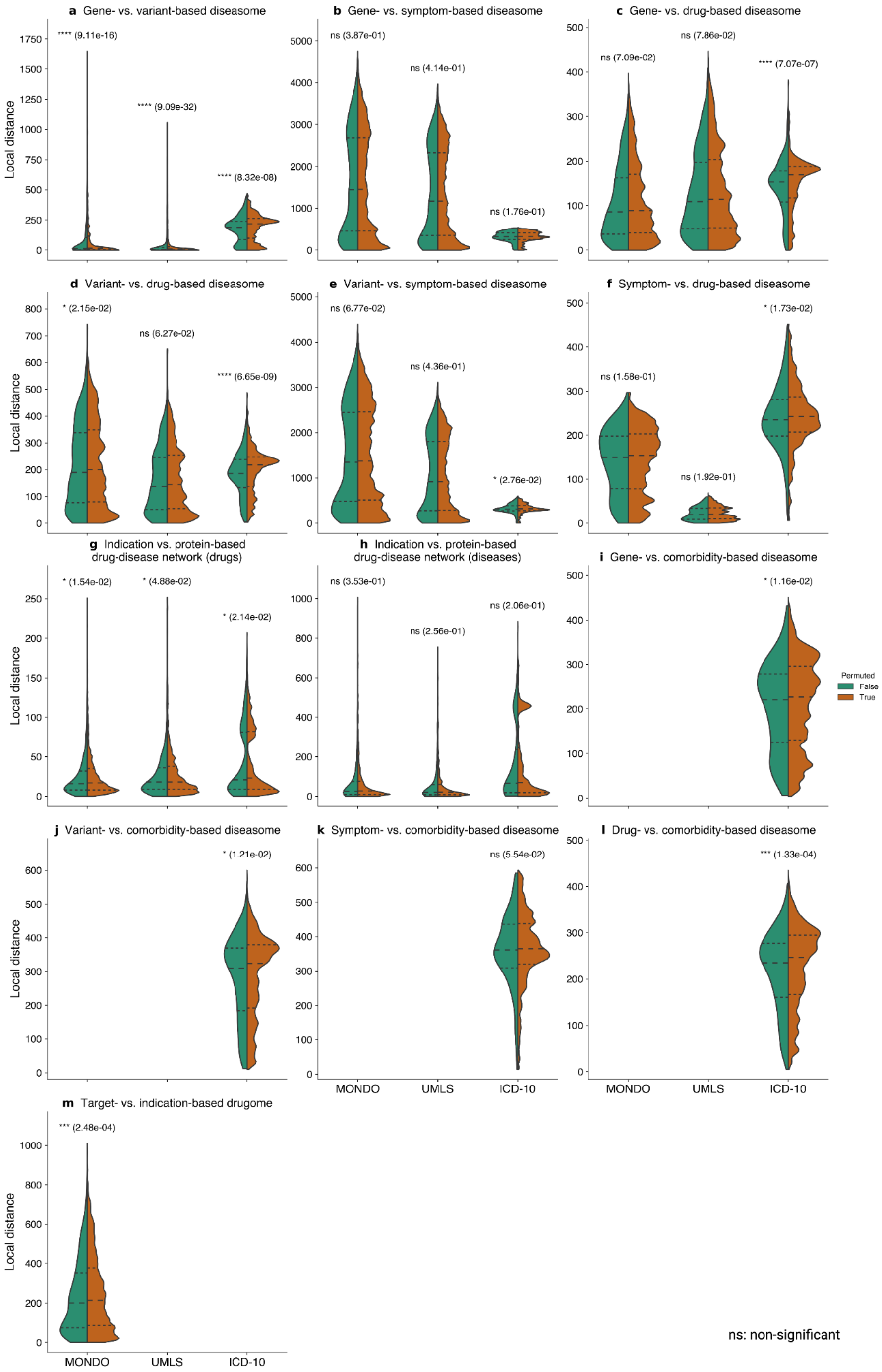

Supplementary Figure 7. Pairwise similarities between networks, global view on local distances, uniform edge edit costs. Local GEDs (of all nodes) between a pair of networks for the original vs. permuted network and corresponding Mann-Whitney U *P*-values (one-sided, unadjusted). (a-f) Similarities between diseasomes in MONDO, UMLS, and ICD-10 vocabularies. (g-h) Indication- vs. protein-based drug-disease network in MONDO, UMLS, and ICD-10 vocabularies (separately for drugs and diseases). (i-l) Comorbidity-based vs. other diseasomes in ICD-10 vocabularies. (m) Target- vs. indication-based drugomes.

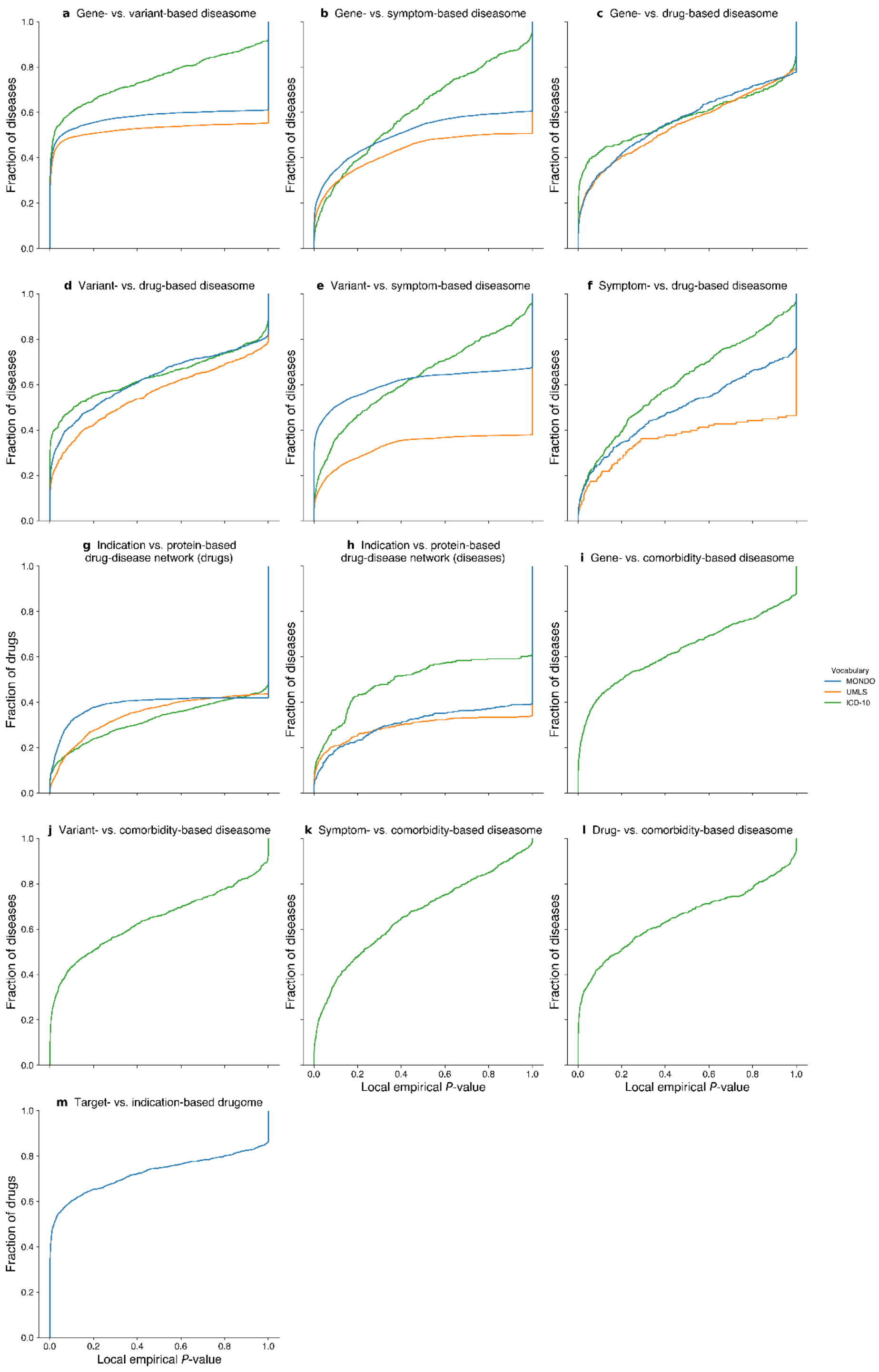

Supplementary Figure 8. Pairwise similarities between networks, local view on local GEDs, uniform edge edit costs. Local empirical *P*-values (one-sided, unadjusted) computed from GEDs on a pair of networks for the original vs. permuted network. (a-f) Similarities between diseasomes in MONDO, UMLS CUI, and ICD-10 vocabularies. (g-h) Indication- vs. protein-based drug-disease network in MONDO, UMLS CUI, and ICD-10 vocabularies (separately for drugs and diseases). (i-l) Comorbidity-based vs. other diseasomes in ICD-10 vocabularies. (m) Target- vs. indication-based drugomes.

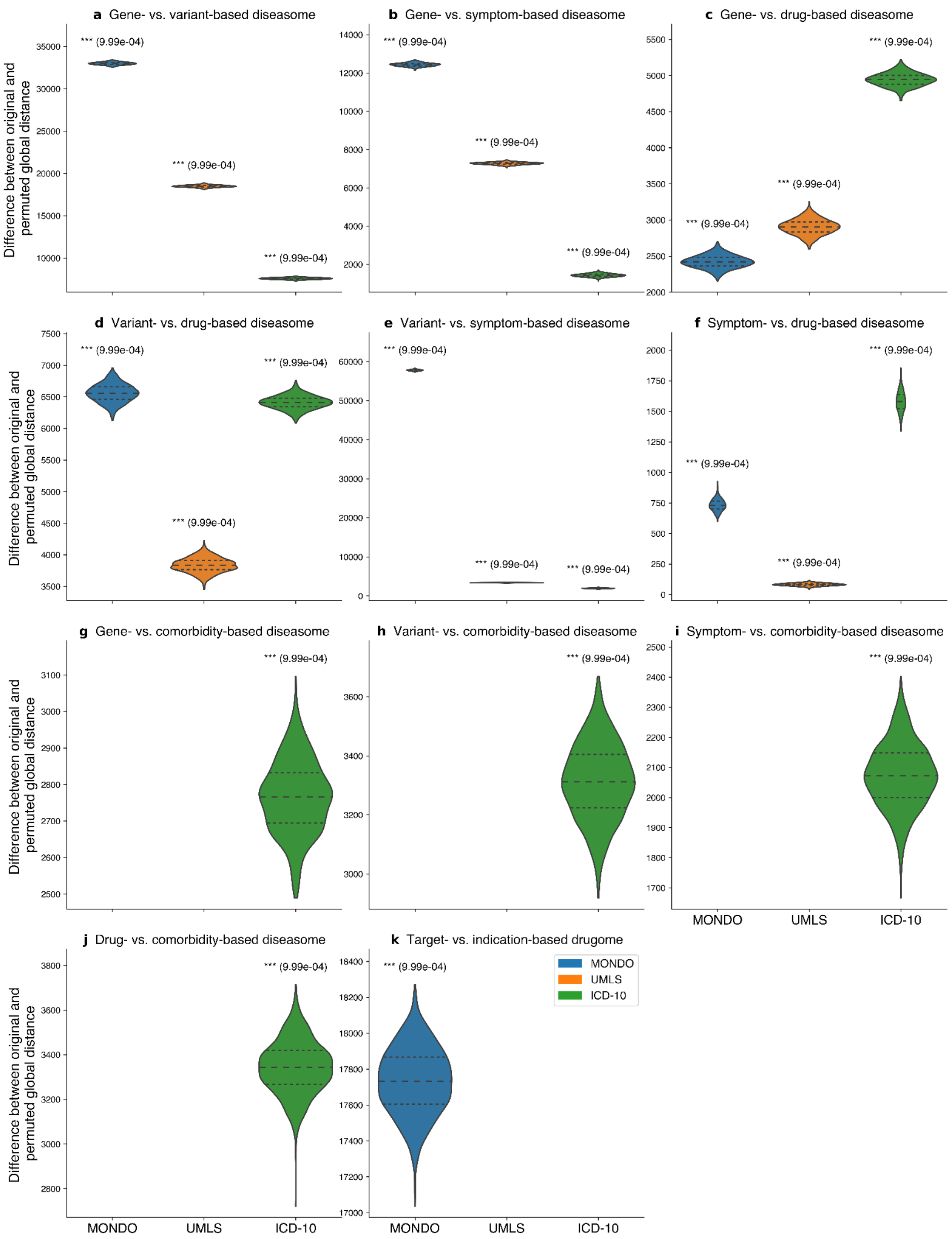

Supplementary Figure 9. Pairwise similarities between networks, global distances, weight-based edge edit costs. Differences of global GEDs between the original and permuted network, and corresponding global empirical *P*-values (one-sided, unadjusted). (a-f) Similarity between diseasomes in MONDO, UMLS CUI and ICD-10 vocabularies. (g-j) Comorbidity-based vs. other diseasomes in ICD-10 vocabularies. (k) Target- vs. indication-based drugomes. All obtained global empirical *P*-values were at the lower resolution limit of our permutation tests with 1,000 randomized network pairs.

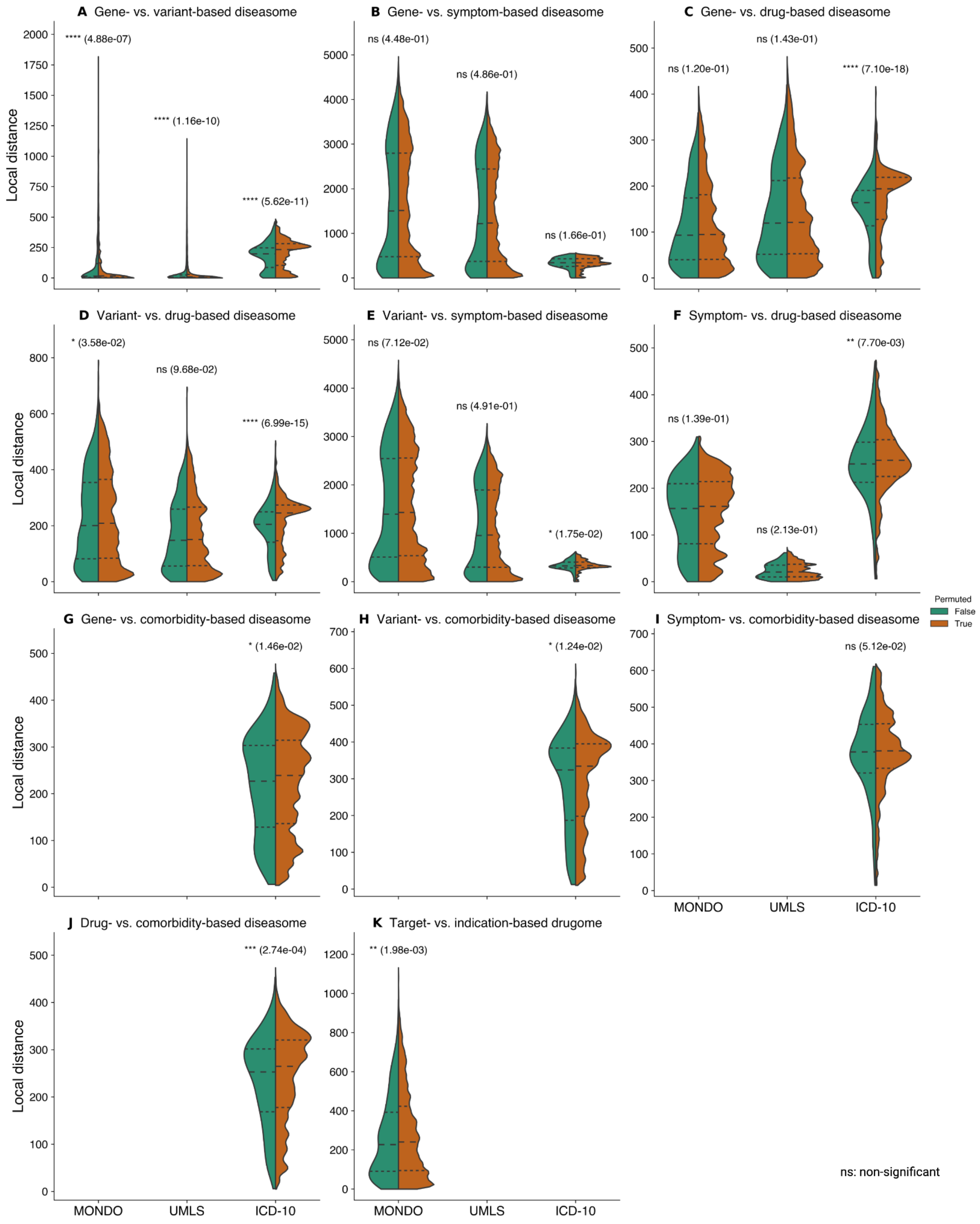

Supplementary Figure 10. Pairwise similarities between networks, global view on local distances, weight-based edge edit costs. Local GEDs (of all nodes) between a pair of networks for the original vs. permuted network and corresponding Mann-Whitney U *P*-values (one-sided, unadjusted). (a-f) Similarity between diseasomes in MONDO, UMLS CUI, and ICD-10 vocabularies. (g-j) Comorbidity-based vs. other diseasomes in ICD-10 vocabulary. (k) target- vs. indication-based drugomes.

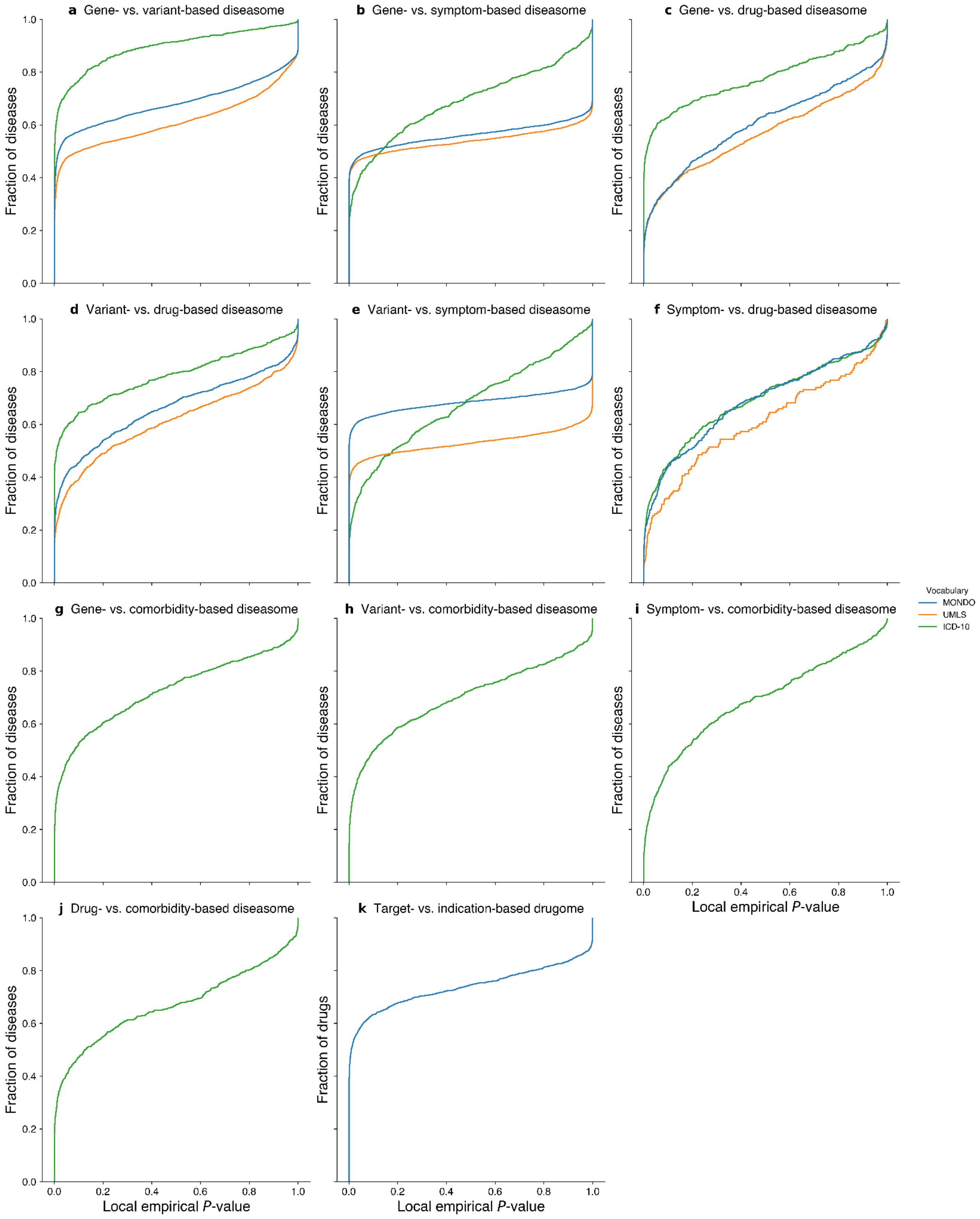

Supplementary Figure 11. Pairwise similarities between networks, local view on local distances, weight-based edge edit costs. Local empirical *P*-values (one-sided, unadjusted) computed from GEDs on a pair of networks for the original vs. permuted network. (a-f) Similarity between diseasomes in MONDO, UMLS CUI, and ICD-10 vocabularies. (g-j) Comorbidity-based vs. other diseasomes in ICD-10 vocabularies. (k) Target- vs. indication-based drugomes.

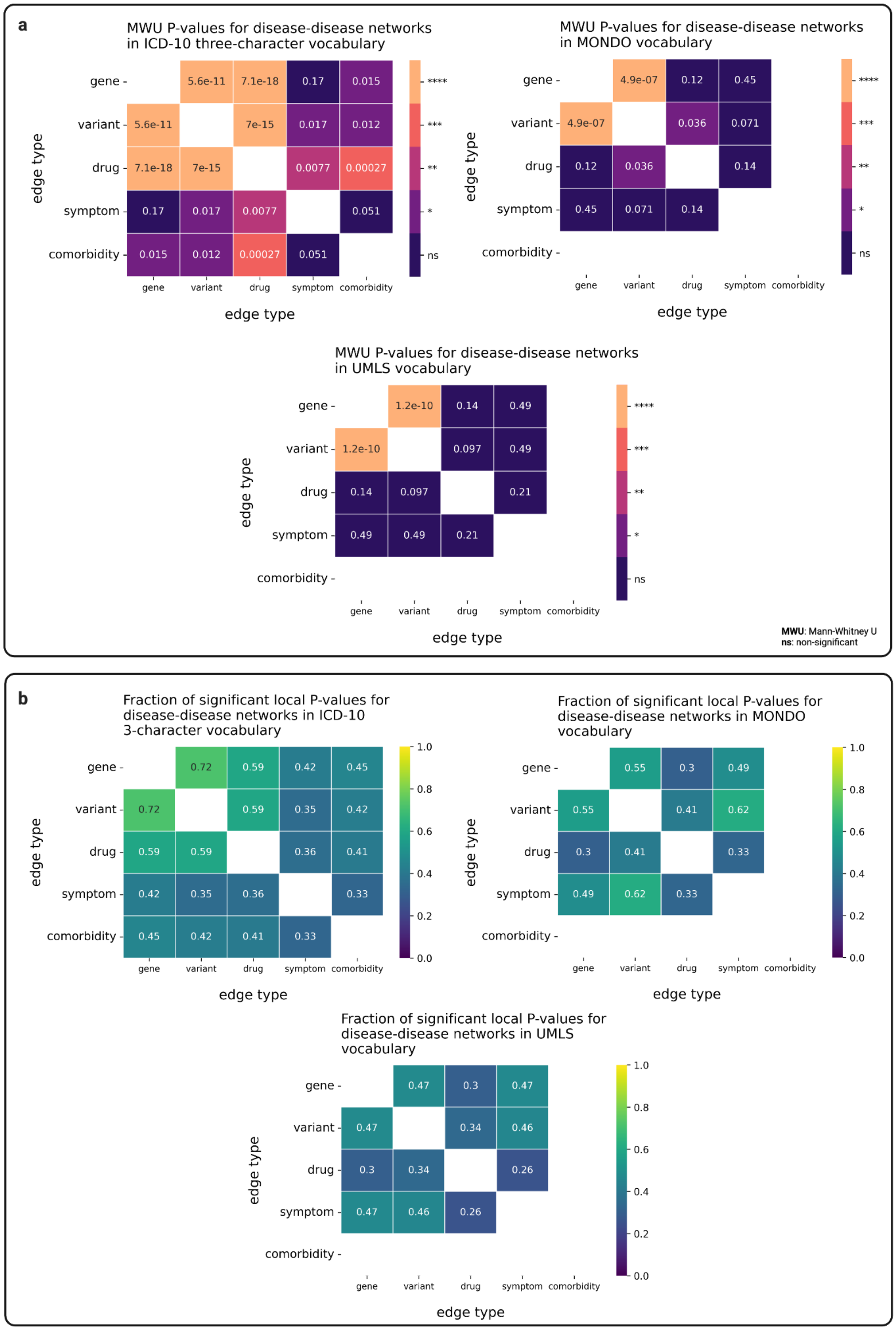

Supplementary Figure 12. Overview of local-scale analyses, weight-based edge edit costs. (a) Mann-Whitney U *P*-values (one-sided, unadjusted) computed from local GEDs with the level of their significance. (b) Fraction of significant local empirical *P*-values (one-sided, unadjusted) at 0.05 level computed from local GEDs on a pair of networks for the original vs. permuted network.

Supplementary Table 1. Properties of constructed networks. Drugomes were constructed only in MONDO vocabulary and the comorbidity-based diseasome only in ICD-10 vocabulary (since Estonian Biobank uses ICD-10 codes, mapping the comorbidities to the MONDO vocabulary was impossible).

Network	# Nodes	# Edges	# CCs	Size of LCC	Density	# Isolated nodes
**MONDO vocabulary**						
Gene-based diseasome	8,284	118,726	1,571	6,598	0.00346	1,483
Variant-based diseasome	8,759	677,241	1,401	7,162	0.01766	1,250
Symptom-based diseasome	9,926	10,982,517	43	9,883	0.22296	41
Drug-based diseasome	1,772	129,788	42	1,724	0.08271	36
Target-based drugome	5,878	272,010	164	5,619	0.01575	108
Indication-based drugome	2,249	194,959	42	2,195	0.07712	34
Protein-based drug-disease network	1,629 dr[1]<br>607 dis[2]	37,360	1	2,236	0.03778	0
Indication-based drug-disease network	2,249 dr<br>1,772 dis	15,800	1	4,021	0.00396	0
Drug-protein-protein-disease	7,473 dis<br>5,878 dr<br>24,757 pr	422,617	52	37,967	0.00066	0
Disease-gene-gene-disease	8,284 dis<br>18,113 g	371,385	810	24,768	0.00118	0
**ICD-10 vocabulary (3-character codes)**						
Gene-based diseasome	755	45,671	28	728	0.16045	27
Variant-based diseasome	894	101,554	22	872	0.25441	20
Symptom-based diseasome	735	150,618	3	733	0.37165	2
Drug-based diseasome	714	60,832	8	705	0.23899	5
Comorbidity-based diseasome	1,114	122,030	1	1,114	0.19684	0
Protein-based drug-disease network	1,588 dr<br>382 dis	63,690	1	1,970	0.10499	0
Indication-based drug-disease network	1,950 dr<br>714 dis	14,299	8	2,640	0.01027	0
**UMLS CUI vocabulary**						
Gene-based diseasome	8,475	126,267	1,675	6,520	0.00352	1,481
Variant-based diseasome	14,155	879,830	1,912	12,018	0.00878	1,754
Symptom-based diseasome	7,308	5,287,294	46	7,263	0.19803	45
Drug-based diseasome	1,752	106,567	70	1,678	0.06948	65
Protein-based drug-disease network	5,155 dr<br>3,741 dis	161,982	14	8,833	0.00840	0
Indication-based drug-disease network	2,349 dr<br>1,752 dis	14,703	70	3,943	0.00357	0

[1]dr: drug
[2]dis: disease
