# Supplementary material for: Lacking mechanistic disease definitions and corresponding association data hamper progress in network medicine and beyond
Source: Nat Commun. 2023 Mar 25;14:1662. doi: 10.1038/s41467-023-37349-4 (PMC10039912; doi:10.1038/s41467-023-37349-4)
Supplement: Supplementary file 3 — Reporting Summary [file 41467_2023_37349_MOESM3_ESM.pdf]

nature portfolio

Corresponding author(s): Sepideh Sadegh , David B. Blumenthal

Last updated by author(s): 02/01/2023

Reporting Summary

Nature Portfolio wishes to improve the reproducibility of the work that we publish. This form provides structure for consistency and transparency in reporting. For further information on Nature Portfolio policies, see our Editorial Policies and the Editorial Policy Checklist.

Statistics

For all statistical analyses, confirm that the following items are present in the figure legend, table legend, main text, or Methods section.

n/a	Confirmed	
x		The exact sample size (*n*) for each experimental group/condition, given as a discrete number and unit of measurement
x		A statement on whether measurements were taken from distinct samples or whether the same sample was measured repeatedly
	x	The statistical test(s) used AND whether they are one- or two-sided<br>*Only common tests should be described solely by name; describe more complex techniques in the Methods section.*
x		A description of all covariates tested
	x	A description of any assumptions or corrections, such as tests of normality and adjustment for multiple comparisons
x		A full description of the statistical parameters including central tendency (e.g. means) or other basic estimates (e.g. regression coefficient) AND variation (e.g. standard deviation) or associated estimates of uncertainty (e.g. confidence intervals)
	x	For null hypothesis testing, the test statistic (e.g. F, t, r) with confidence intervals, effect sizes, degrees of freedom and P value noted<br>*Give P values as exact values whenever suitable.*
x		For Bayesian analysis, information on the choice of priors and Markov chain Monte Carlo settings
x		For hierarchical and complex designs, identification of the appropriate level for tests and full reporting of outcomes
x		Estimates of effect sizes (e.g. Cohen's *d*, Pearson's *r*), indicating how they were calculated

Our web collection on statistics for biologists contains articles on many of the points above.

Software and code

Policy information about availability of computer code

Data collection	The GraphSimQT source code is publicly available under the GPL3 license in the Github repository: https://github.com/repotrial/graphsimqt
Data analysis	All network analysis approaches underlying this study are implemented in a Python package called GraphSimQT (“graph similarity quantification tool”), which is openly available on GitHub (https://github.com/repotrial/graphsimqt) under the terms of the GNU General Public License, Version 3.<br>The source code of the frontend and the backend of GraphSimViz is available at https://github.com/repotrial/GraphSimViz-frontend and https://github.com/repotrial/GraphSimViz-backend, respectively.

For manuscripts utilizing custom algorithms or software that are central to the research but not yet described in published literature, software must be made available to editors and reviewers. We strongly encourage code deposition in a community repository (e.g. GitHub). See the Nature Portfolio guidelines for submitting code & software for further information.

Data

Policy information about availability of data

All manuscripts must include a data availability statement. This statement should provide the following information, where applicable:

- Accession codes, unique identifiers, or web links for publicly available datasets
- A description of any restrictions on data availability
- For clinical datasets or third party data, please ensure that the statement adheres to our policy

All networks underlying the findings of this study are available at https://github.com/repotrial/graphsimqt/tree/main/data/graphs.
The following public databases were used to generate the networks: IID (http://iid.ophid.utoronto.ca/), DrugBank (https://go.drugbank.com/), DrugCentral (https://drugcentral.org/), CTD (http://ctdbase.org/), DisGeNET (https://www.disgenet.org/), OMIM (https://omim.org/), UniProt (https://www.uniprot.org/), MONDO (https://MONDO.monarchinitiative.org/), NeDRex (https://nedrex.net/), and HPO (https://hpo.jax.org/app/).
Version numbers of all used databases can be found in an AIMe report for our study (https://aime.report/6bdnlg). The comorbidity-based diseasome was constructed based on data provided by the Estonian Biobank (https://genomics.ut.ee/en/content/estonian-biobank, available from the Estonian Biobank upon request). The raw comorbidity data are not publicly available as they contain person-sensitive information. To obtain access to raw data, the study needs to be approved by the Estonian Biobank.The Estonian Biobank data was used under data release M11, which is covered by ethics approval 234T-12 Omics for Health. The source data necessary for reproducing this research are provided with this paper (data underlying the plots are available for download at https://api.graphsimviz.net/download_results and https://github.com/repotrial/graphsimqt). Our study is based on public databases (including DisGeNET, OMIM, DrugBank, HPO, and more) which do not contain sex-specific information. Therefore, no sex-specific analyses could be carried out.

Human research participants

Policy information about studies involving human research participants and Sex and Gender in Research.

Reporting on sex and gender	Sex and/or gender was not considered in the study design. For further information see Data Availability section.
Population characteristics	There is no covariate-relevant population characteristics applicable to this study.
Recruitment	No selection step was involved. The comorbidity part of the study is based on the available health records.
Ethics oversight	Estonian Committee on Bioethics and Human Research

Note that full information on the approval of the study protocol must also be provided in the manuscript.

Field-specific reporting

Please select the one below that is the best fit for your research. If you are not sure, read the appropriate sections before making your selection.

☒ Life sciences ☐ Behavioural & social sciences ☐ Ecological, evolutionary & environmental sciences

For a reference copy of the document with all sections, see nature.com/documents/nr-reporting-summary-flat.pdf

Life sciences study design

All studies must disclose on these points even when the disclosure is negative.

Sample size	No sample-size calculation was performed. The only data containing patient-level data is the comorbidity data collected from 139,065 patients (Estonian Biobank data). This study involves essentially no sampling and the entire data available in the Estonian Biobank was used for this study.
Data exclusions	No data from the original datasets were excluded, unless it is specifically described in the Methods section of this article. HPO contains both general and specific terms, we pruned the data by removing very general symptom terms using the existing hierarchy in HPO. More specifically, we removed the terms from the top three levels after decomposing the generated hierarchical phenotype network. From the comorbidity data, we removed diseases with incidence below five, as well as the codes from the ICD-10 chapters XV ("Pregnancy, childbirth and the puerperium"), XVI ("Certain conditions originating in the perinatal period"), XVIII ("Symptoms, signs and abnormal clinical and laboratory findings, not elsewhere classified"), XIX ("Injury, poisoning and certain other consequences of external causes"), XX ("External causes of morbidity and mortality"), XXI ("Factors influencing health status and contact with health services"), and XXII ("Codes for special purposes").
Replication	The analyses were repeated ten times, all results could be replicated.
Randomization	No allocation of participants into groups was applicable to this study. ICD-10 diagnoses were obtained from epicrises, prescriptions and bills to the Health Insurance Fund.<br>Network randomization was done by rewiring edges with node degree preserving. For each pair-wise network comparison we computed empirical P-values based on the background models generated from 1000 randomizations.

Blinding	N/A - The study did not include any direct interaction with human that could influence participants. ICD-10 diagnoses were obtained from epicrises, prescriptions and bills to the Health Insurance Fund.

Reporting for specific materials, systems and methods

We require information from authors about some types of materials, experimental systems and methods used in many studies. Here, indicate whether each material, system or method listed is relevant to your study. If you are not sure if a list item applies to your research, read the appropriate section before selecting a response.

Materials & experimental systems

n/a	Involved in the study
☒	☐ Antibodies
☒	☐ Eukaryotic cell lines
☒	☐ Palaeontology and archaeology
☒	☐ Animals and other organisms
☒	☐ Clinical data
☒	☐ Dual use research of concern

Methods

n/a	Involved in the study
☒	☐ ChIP-seq
☒	☐ Flow cytometry
☒	☐ MRI-based neuroimaging
